# Increased radiosensitivity and radiothermosensitivity of human pancreatic MIA PaCa-2 and U251 glioblastoma cell lines treated with the novel Hsp90 inhibitor NVP-HSP990

**DOI:** 10.1186/1748-717X-8-42

**Published:** 2013-02-28

**Authors:** Dušan Milanović, Elke Firat, Anca Ligia Grosu, Gabriele Niedermann

**Affiliations:** 1 Department of Radiation Oncology, University Hospital Freiburg, Freiburg 79106, Germany

**Keywords:** Radiosensitivity, Radiothermosensitivity, NVP-HSP990

## Abstract

**Background and purpose:**

Heat shock Protein 90 (Hsp90) is a molecular chaperone that folds, stabilizes, and functionally regulates many cellular proteins involved in oncogenic signaling and in the regulation of radiosensitivity. It is upregulated in response to stress such a heat. Hyperthermia is a potent radiosensitizer, but induction of Hsp90 may potentially limit its efficacy. Our aim was to investigate whether the new Hsp90 inhibitor NVP-HSP990 increases radiosensitivity, thermosensitivity and radiothermosensitivity of human tumor cell lines.

**Material and methods:**

U251 glioblastoma and MIA PaCa-2 pancreatic carcinoma cells were used. To determine clonogenic survival, colony forming assays were performed. Cell viability and proliferation were assesed by Trypan blue staining. Cell cycle and apoptosis analyses were performed by flow cytometry. DAPI staining was used to detect mitotic catastrophe.

**Results:**

NVP**-**HSP990 increased the thermosensitivity, radiosensitivity and radio-thermosensitivity of both cell lines in clonogenic assays. 72 hours after irradiation with 4 Gy, a significant reduction in cell number associated with considerable G2/M acumulation and mitotic catastrophe as well as cell death by apoptosis/necrosis was observed.

**Conclusions:**

Treatment with NVP**-**HSP990 strongly sensitized U251 and MIA PaCa-2 cells to hyperthermia and ionizing radiation or combination thereof through augmentation of G2/M arrest, mitotic catastrophe and associated apoptosis.

## Introduction

Heat shock protein 90 (Hsp90) is an evolutionary conserved molecular chaperone which under physiological conditions participates in protein folding, intracellular transport, maintenance and degradation of proteins. Proteins, which are activated and stabilized by Hsp90, are referred to as “clients”. A lot of them are crucial for constitutive cell signaling and adaptive responses to stress [1]. Hsp90 is expressed at 2–10-fold higher levels in tumor tissue than in normal tissue [2]. Its most important function is to protect mutated and overexpressed oncoproteins from misfolding and degradation. It has been recognized to be essential for the stability and function of a wide variety of kinases such as EGFR, Erb-B2, Akt, BCR-ABL, VEGFR2 involved in cell cycle regulation, survival and oncogenic signaling [3]. These proteins play also critical roles in the regulation of radiosensitivity [4-6]. Thus, the inhibition of Hsp90 may represent an attractive therapeutic strategy not only reducing basal survival of tumor cells but also increasing their radiosensitivity.

Heat is a very potent radiosensitizer *in vitro* and *in vivo*[7,8]. Clinical studies have shown that the combination of conventional radiation therapy with hyperthermia leads to significantly better tumor control [9]. Hsp90 is heat-inducible in normal and in tumor cells [10]. This heat induction of Hsp90 could limit desirable effects of ionizing radiation (IR) via activation of “client proteins” which may contribute to radioresistance as mentioned above. Theoretically, small molecules designed to inhibit Hsp90 might increase the effect of hyperthermia followed by enhanced radiosensitivity.

NVP-HSP990 is a novel, highly potent orally available 2-aminothienopyrimidine class, non-geldanamycin based Hsp90 inhibitor [11]. It has been shown that treatment of established cell lines from different tumor entities with the non*-*geldanamycin based Hsp90 inhibitors NVP-BEP800 and NVP-AUY922 increase their sensitivity towards IR [12].

Glioblastoma multiforme and pancreatic carcinoma represent tumors which are resistant to conventional radiochemotherapeutical regimes and despite intensive research and development of new targeted therapies prognosis of patients with these tumors remains poor [13,14], indicating the need for new therapeutic approaches.

Here we investigated whether treatment with the Hsp90 inhibitor NVP-HSP990 and hyperthermia enhance thermal sensitivity and consequently radiosensitivity of U251 glioma and MIA PaCa-2 pancreatic carcinoma cells.

## Materials and methods

### Cell culture and reagents

The U251 human glioblastoma and pancreatic carcinoma MIA PaCa-2 cell line were obtained from the tumor bank of the National Cancer Institute (NCI), Frederick, Maryland. Cells were grown as a monolayer in RPMI-1640 culture medium supplemented with 10% foetal bovine serum (FBS; Biochrom, Berlin, Germany) at 37°C under 8.5% CO_2._ NVP-HSP990 was kindly provided by Novartis Institutes for Biomedical Research (Basel, Switzerland). The drug was dissolved in DMSO and stock solutions were stored at −20°C.

### Hyperthermia

Hyperthermia was provided by a cell incubator (Heraeus, type Heracell), reaching temperatures of up to ~55°C with a precision of 0.1°C. The temperature of 37°C was chosen as control. Before starting a hyperthermia, the temperature was controlled with 3 thermocouples from the interstitial hyperthermia device (Academic Ziekenhuis Utrecht, The Netherlands), which were inserted in needles and placed in the cell culture flasks filled with 5 ml culture media. Each thermocouple has 7 different points from which the temperature was measured. When the average temperature value in the incubator reached 42°C, the cells were incubated for 1 hour and afterwards replaced to 37°C.

### Hyperthermia and drug treatment

To investigate the effect of NVP-HSP990 on colony formation as a sole compound, cells were treated with increasing drug concentrations (0.01, 0.02, 0.05 and 0.1 μM). In combination treatment with hyperthermia, the cells were treated with the same drug concentrations. Immediately after adding the drug, the cells were incubated for 1 hour at 42°C and afterwards replaced to 37°C. 24 hours later, the cells were trypsinised and 100 cells were plated for colony forming assay (CFA) without drug using 25 cm^2^ tissue flasks (Falcon Becton-Dickinson, Germany).

### γ-irradiation

Irradiation was performed at room temperature using a Gammacell 40 ^137^Cs laboratory irradiator. After irradiation, the cells were recovered in growth medium for 24 hours until harvest.

### Combined treatment with NVP-HSP990, hyperthemia and IR

**Figure 1:**
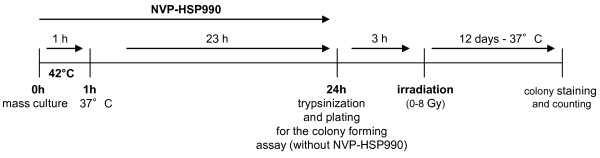
Schematic diagram illustrating the treatment schedule for cell survival experiments.

200.000 U251 cells were seeded in 25 cm^2^ tissue culture flasks and 24 hours later treated with 0.02 or 0.1 μM NVP-HSP990. Control cells were treated in parallel with respective concentrations of DMSO as a vehicle control. 24 hours after start of treatment with NVP-HSP990, the cells were trypsinised and plated for CFA without drug. In experiments, where hyperthermia was used, the cells were treated with 0.05 μM NVP-HSP990. Immediately after adding the drug, cells were icubated at 42°C for 1 hour and afterwards replaced to 37°C. Figure 1 shows the treatment schedule for these experiments.

### Colony forming assay (CFA)

After allowing the cells to attach to the petri dish, the cells were irradiated with 0, 2, 4, 6 or 8 Gy without NVP-HSP990. 12 days after seeding, the colonies were fixed with methanol/acetic acid (3:1) and stained with crystal violet dye (1%). The number of colonies containing at least 50 cells was determined, and the surviving fractions were calculated. The curves were normalised to SF1 (100% cell survival). The surviving fractions were calculated using the plating efficiency for each treatment group (42°C, NVP-HSP990 or 42°C + NVP-HSP990 combination). Plating efficiency and surviving fractions were determined for each cell line and treatment. Cell survival curves were fitted by the linear-quadratic model SF = exp[−(αD + βD^2^)].

### Assessment of cell proliferation and viability by Trypan blue exclusion and FACS analysis

To determine the number of viable cells, trypan blue exclusion tests were conducted. To assess induction of apoptosis and global cell death, annexin-V and propidium-iodide (PI) double staining was performed using the Annexin-V Apoptosis Detection Kit (Miltenyi Biotec). Briefly, U251 and MIA PaCa-2 cells were treated with 0.05 μM NVP-HSP990. Immediately after adding the drug, the cells were incubated for 1 hour at 42°C and then replaced to 37°C. 24 hours later, the growth medium was replaced and the cells were irradiated with a single dose of 4 Gy. 24 and 72 hours later, the cells were stained with annexin V-FITC and PI and analyzed by FACS (FMT 500) from Beckman Coulter.

### Cell cycle analyses

Exponentially growing U251 and MIA PaCa-2 cells were treated and fixed 8, 24 and 48 hours later with 70% ethanol. After storage at −20°C overnight, the cells were washed and incubated with PI (50 μg/mL) and RNase (100 μg/mL) for 2 h at 4°C. After washing, the cells were analyzed for DNA content by flow cytometry.

### Assessment of mitotic catastrophe

U251 cells were treated as described. 3 or 5 days later, the cells were fixed, stained with 4'-6-diamidino-2-phenylindole (DAPI) and analysed under an Olympus BX41 fluorescence microscope equipped with a digital camera CC-12 soft imaging system (U-CMAD3, Olympus). For each assessment of the extent of mitotic catastrophe, 200 nuclei were examined.

### Statistical analyses

The Mann–Whitney *U* Test and Kruskal-Wallis analysis of variance were used to compare quantification data. Statistical analysis was conducted with Statistical Package for Social Sciences software (SPSS Inc.). We used a 2-sided test with significance level of 0.05 for all statistical analyses. Synergy was calculated by the fractional product method that allows an evaluation of synergy at a defined level of effect [15].

## Results

### The effect of hyperthermia and NVP-HSP990 on clonogenic survival of U251 and MIA PaCa-2 cells

**Figure 2:**
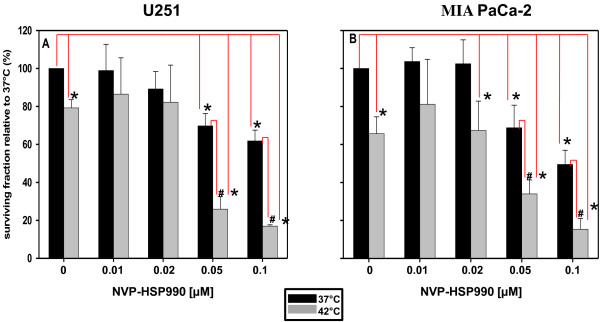
**Colony forming assay after exposure of U251 (A) or MIA PaCa-2 (B) cell lines to different concentrations of NVP-HSP990 alone at 37°C (black bars) or in combination with hyperthermia (grey bars). **Triplicate data from two experiments were averaged and normalised against non-treated controls (DMSO). Colonies containing at least 50 cells were scored. All data are obtained from 3 independent experiments, each performed in triplicate. The results were considered to be statistically significant when P < 0.05 (“comparison to control”-*, “comparison to drug only”-#).

Treatment with 0.01 or 0.02 μM NVP-HSP990 (Figure 2A and 2B) did not influence colony formation while the treatment with 0.05 μM NVP-HSP990 slightly but significantly reduced colony numbers in both cells lines compared to untreated controls (U251 p = 0.0022, MIA PaCa-2 p = 0.042). Similar results were observed if cells were treated with 0.1 μM NVP-HSP990. Sole heat treatment caused modest but also significant reduction of colony number in both cell lines (U251 p = 0.003, MIA PaCa-2 p = 0.042). The treatment of U251 cells with 0.05 μM NVP-HSP990 at 42°C markedly reduced colony numbers compared to either treatment alone (NVP-HSP990 vs. NVP-HSP990 at 42°C p = 0.00034). Similar results were observed if the cells were treated with 0.1 μM NVP-HSP990 at 42°C. In case of MIA PaCa-2 cells, incubation at 42°C in the presence of 0.05 or 0.1 μM NVP-HSP990 caused significant reduction of colony formation compared to treatment with NVP-HSP990 alone (p = 0.00034 for both concentrations).

### The effect of NVP-HSP990 on cellular radiosensitivity assesed by CFA

**Figure 3:**
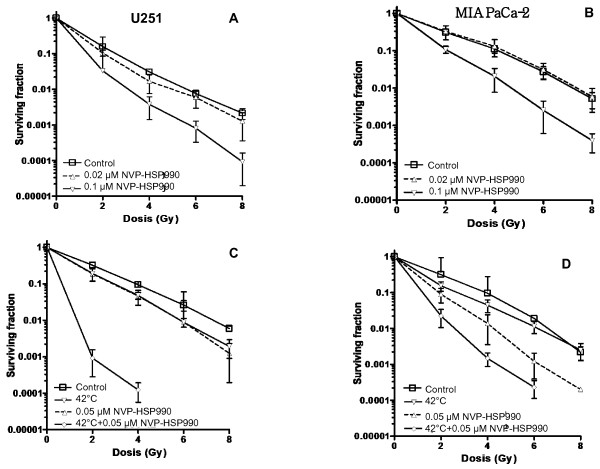
**Influence of NVP-HSP990 and hyperthermia on the radiosensitivity of U251 and MIA PaCa-2 cells (A and B). **The cells were incubated with 0.02 or 0.1 μM NVP-HSP990 for 24 h. Thereafter, growth media containing NVP-HSP990 were replaced with fresh medium and the cells were irradiated with single IR doses ranging between 2 and 8 Gy (**C **and **D**). In the case of the combined treatment with NVP-HSP990 and hyperthermia, U251 cells (**C**) or MIA PaCa-2 cells (**D**) were simultaneously treated with 0.05 μM NVP-HSP990 and 42°C for 1 hour, then replaced to 37°C for the next 23 hours and then irradiated as described above. Colony forming efficiency was determined 12 days later and colonies containing at least 50 cells were scored. Data show mean values and range of 2 independent experiments each plated in triplicate.

The influence of NVP-HSP990 on the radiosensitivity of U251 and MIA PaCa-2 cells was also determined by CFA. Based on the data shown in Figure 2A and 2B, the cells were pretreated with 0.02 or 0.1 μM NVP-HSP990 for 24 h, then seeded as single cells and exposed to X-ray doses up to 8 Gy. The radiosensitivity was determined by CFA. Pretreatment with 0.1 μM NVP-HSP990 markedly increased radiosensitivity of both U251 (Figure 3A) and MIA PaCa-2 (Figure 3B) cells at all dose levels while pretreatment with 0.02 μM NVP-HSP990 did not change radisensitivity of both cell lines.

### Combined treatment with NVP-HSP990 and hyperthermia strongly increases the radiosensitivity of U251 and MIA PaCa-2 cells

The influence of the combined treatment with NVP-HSP990 and hyperthermia on the radiosensitivity of both cell lines was also analysed by CFA. While treatment with 0.05 μM NVP-HSP990 or heating with 42°C for 1 hour had only a modest effect on radiosensitivity of U251 cells (Figure 3C), the combination treatment caused a potent radiosensitization. In case of irradiation with 6 or 8 Gy, no colony formation was detected anymore. Treatment of the MIA PaCa-2 cells (Figure 3D) with 0.05 μM NVP-HSP990 had a more pronounced radiosensitizing effect in comparison to U251 cells. Heating of these cells caused further radiosensitization at any irradiation dose. At a dose of 8 Gy, no colony formation was observed anymore.

### Effect of treatments on proliferation and apoptosis in U251 and MIA PaCa-2 cells

**Figure 4:**
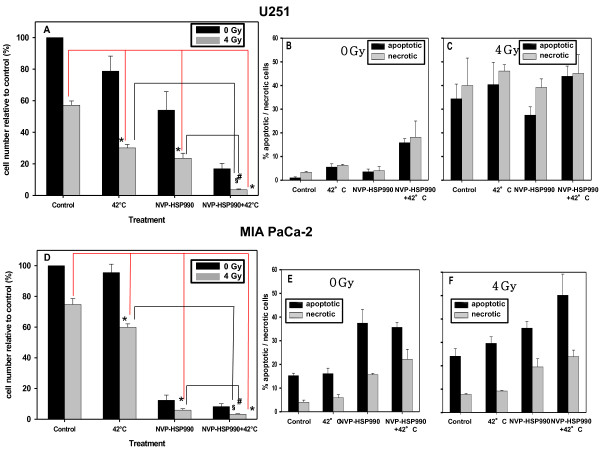
**The effects of treatment with NVP-HSP990, hyperthermia and 4 Gy IR on cell proliferation and cell death of U251 (A, B, C) and MIA PaCa-2 cells (D, E, F). **In figure **A **and **D **black bars represent non-irradiated cells while grey bars represent cells which were irradiated with a single dose of 4 Gy. The cells were treated with 0.05 μM NVP-HSP990 and 42°C for 1 hour and then replaced to 37°C for the next 23 hours in the presence of the inhibitor. Thereafter the culture medium was replaced with fresh medium without inhibitor and the cells were irradiated with a single dose of 4 Gy. 72 hours later, the cells were stained with trypan blue to determine the number of viable cells (**A**, **D**). Mean value and SEM from 3 independent experiments is shown. To detect the percentage of apoptotic/necrotic cells, cells were stained with FITC-conjugated anti-Annexin V antibody and propidium iodide (**B**, **C**, **E**, **F**). Black bars represent apoptotic while grey bars necrotic cells. Mean values and range of 2 independent experiments are shown.

Trypan blue exclusion staining showed that the strongest reduction in viable cell numbers was indeed achieved in the case of triple combinations (only 5% of the cells survived compared to untreated controls) in U251 (Figure 4A) and MIA PaCa-2 cells (Figure 4D) (p = 0.04935 for both cell lines). All other treatments also caused significant antiproliferative effects. MIA PaCa-2 cells were more sensitive to treatment with NVP-HSP990 alone compared to U251 cells. In the case of U251 cells, the effects of the double combination of NVP-HSP990 plus hyperthermia (calculated 43%, observed 16% surviving cells) and the triple combination including IR (calculated 4.1%, observed 3.64% surviving cells) were synergistic or additive, respectively, compared to the individual treatments (Figure 4A). In the case of MIA PaCa-2 cells these combinations did not synergistically enhance the effect of NVP-HSP990 alone, which was very strong. Analysis of annexin-V/PI -staining by flow cytometry showed that treatment of U251 cells with 42°C or NVP-HSP990 as sole therapeutic modalities did not cause remarkable increase in the number of apoptotic/necrotic cells while combined treatment had a stronger effect (Figure 4B). Irradiation with 4 Gy caused a considerable increase in the number of apoptotic/necrotic cells independently of the treatment (Figure 4B). In the case of MIA PaCa-2 cells annexin-V/PI -staining revealed that NVP-HSP990 alone caused a higher apoptotic/necrotic percentage in comparison to U251 cells and that additionally heating of these cells did not augment the effect of NVP-HSP990 (Figure 4E). The most pronounced increase in numbers of apoptotic and dead cells in MIA PaCa-2 cells was observed after treatment with the triple combination (Figure 4F).

### Cell cycle alterations in U251 and MIA PaCa-2 cells

**Figure 5:**
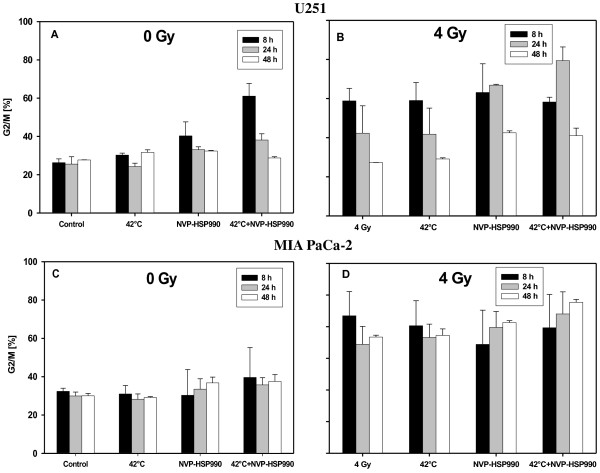
**Effect of different treatments on G2/M arrest of U251 (A, B) and MIA PaCa-2 cells (C, D). **The cells were heated for 1 h with 42°C and treated with 0.05 μM NVP**-**HSP990 for 24 h as a sole modality (**A**, **C**) or in combination with irradiation with 4 Gy (**B**, **D**). The percentage of cells accumulating in G2/M is shown. Mean values and range of 2 independent experiments are shown.

Figure 5A shows that U251 cells which were heated with 42°C or treated with NVP-HSP990 did not show differences in the number of cells accumulating in the G2/M phase. 8 hours after treatment with hyperthermia and NVP-HSP990, increased numbers of cells accumulated in G2/M. After 24 or 48 hours there was no difference compared to untreated cells. 8 hours after irradiation with 4 Gy (Figure 5B) an increased number of cells accumulated in G2/M, while 48 hours after irradiation the cell number dropped to the level of untreated cells. A similar result was obtained when the cells were heated before irradiation. In the case of the triple combination, maximal G2/M-arrest was reached only after 24 hours. In MIA PaCa-2 cells only irradiation with 4 Gy caused an increased number of cells accumulating in G2/M.

### Effect on mitotic catastrophe in U251 cells

**Figure 6:**
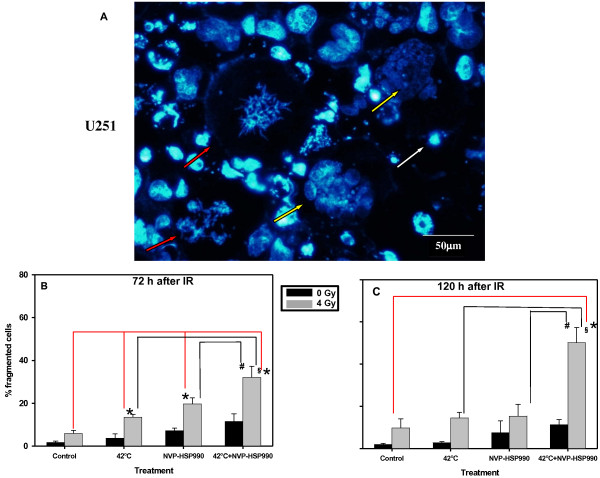
**Effect of treatments on mitotic catastrophe (A). **Representative image showing aberrant mitotic figures and increased numbers of multinucleated and apoptotic cells observed in U251 cell culture treated with NVP-HSP990, hyperthermia and γ-IR. 72 hours or 120 hours after γ-IR, DAPI was used to stain the DNA. Yellow arrowheads indicate multinucleated, white arrowheads apoptotic (with condensed DNA) and red arrowheads cells with aberrant mitotic figures. Quantification was performed after 72 h (**B**) or 120 hours (**C**) after IR. 200 cells were counted per sample. Mean value and SEM of 3 independent experiments are shown.

3 or 5 days after a 4 Gy irradiatiation, DAPI stainings were performed and the morphology of the cells and their nuclei were microscopically analysed (Figure 6A-C). Hyperthermia or NVP-HSP990 inhibitor alone did not significantly increase the percentage of cells with signs of mitotic catastrophe (micro- or multinucleated cells), whereas the double combination of the two slightly increased it. In γ-irradiated cells, any additional treatment caused an increase in the number of cells undergoing mitotic catastrophe. The strongest increase (ca. 60% of cells) was found in cultures treated with the triple combination (p = 0.04935). Additive effects were observed after 3 days and 5 days in double and triple combinations (double combination after 5 days expected 9.9% fragmented cells, observed 11.33%, triple combination expected 34.5%, observed 50.16% fragmented cells).

## Discussion

The treatmant of patients with glioblastoma and pancreatic carcinoma remains a challenge. In the present study, we found that pretreatment with the novel Hsp90 inhibitor NVP-HSP990 strongly sensitizes U251 glioma and MIA PaCa-2 pancreatic carcinoma cells to hyperthermia and IR and particulary to the combination thereof. The triple combination caused a significant reduction in cell number which was associated with a morphological alterations typical of mitotic catastrophe and apoptosis.

There is a lot of experimental [16-18] and clinical evidence that hyperthermia can increase the effectiveness of other conventional treatments such chemotherapy [19] or especially radiotherapy [20,21]. The exact mechanism how hyperthermia increases radiosensitivity is still not completely elucidated but it has been proposed that hyperthermia may interfere with radiation-induced DNA damage [22]. On the other hand, heat shock proteins mediate resistance to hyperthermia [23]. It has been shown that inhibition of Hsp90 with geldanamycin causes delayed and impaired recovery from heat shock [24]. In HEK293 cells, specific inhibition of Hsp90 together with short term exposure (20–60 min) to 42°C was highly cytotoxic, through accelerated degradation of Cdc25A [25], a member of the CDC25 family of phosphatases which is specifically degraded in response to DNA damage [26]. These findings support the hypothesis that the antineoplastic effect of hyperthermia could be potentiated by concurrent inhibition of Hsp90. In our experimental conditions, we observed that U251 and MIA PaCa-2 cells which were incubated for 1 hour at 42°C and concurrently treated with NVP-HSP990 showed a significantly lower capability to form colonies in comparison to cells which were treated with only one of the two modalities.

The effects of combined hyperthermia and irradiation treatment depend on many different factors such a heating temperature, heating time, sequence and time interval between the two modalities [27]. Despite intensive research, it is still not clear wheather hyperthermia before or after irradiation causes a more pronounced enhancement of radiation damage. Probably, this effect is cell-type specific. In the case of concurrent irradiation and hyperthermia, maximal additive/synergistic effects can be expected while increasing the intervals of time between hyperthermia and IR, regardless of sequence, will abrogate the radiosensitisation induced by hyperthermia [28]. As expected, because of the time interval of 23 hours between hyperthermia and IR, we observed only a weak influence of hyperthermia on radiosensitivity, cell cycle distribution, induction of mitotic catastrophe and apoptosis.

It has been proposed that the radiosensitising effect of Hsp90 inhibitors is caused by degradation of several proteins such a ErbB2, EGFR, Raf-1 and Akt [29,30] which reportedly can influence radioresistance. DNA repair and cell cycle checkpoint activation are other proposed mechanisms by which Hsp90 can influence the DNA damage response to IR [31]. A previous study reported that a 24 h-pretreatment with an Hsp 90 inhibitor similar to NVP-HSP990, NVP-BEP800, caused an increase in radiosensitivity in two glioblastoma, one lung carcinoma and one fibrosarcoma cell line through cell-cycle impairment, increased DNA damage and repair protraction [12]. The authors found that changes in the expression of survival markers (Hsp90, Hsp70, Akt, phospho-Akt, Raf-1 and survivin), an apoptosis-associated protein (cleaved caspase 3) or of the functional p53 status did not significantly contribute to the sensitivity of two out of four tested cell lines to NVP-BEP800 alone or in combination with IR. Another group found that the geldanamycin-based Hsp90 inhibitor 17-dimethylaminoethylamino-17-demethoxygeldanamycin (17-DMAG) enhances radiosensitization of human U251 and MIA PaCa-2 cells [31]. The treatment of the cells with this compound caused a reduction of the expression of Akt, Raf-1 and especially ErbB2. The authors further reported that treatment of DU145 prostate carcinoma cells with 17-DMAG abrogated the G2- and S-phase cell cycle checkpoints and enhanced the radiosensitivity of the cells.

It has been reported that treatment of the human lung adenocarcinoma cell line A549 with KNK437, an benzylidene lactam compound which acts as a heat shock protein inhibitor, causes the enhancement of thermal radiosensitization in mild hyperthermia combined with low dose IR [32]. In the same study, it has been demonstrated that KNK437 caused inhibition of Hsp72 and Hsp27 expression. NVP-HSP990 shows a different mechanism of action; it binds to the NH2-terminal ATP-binding pocket of Hsp90 while KNK437 inhibits synthesis of various heat shock proteins at the mRNA level. KNK437 has also been proposed to induce radioresistance of A-172 human glioblastoma and human squamous cell carcinoma cells [33].

In our experiments, we observed that pretreatment of U251 cells with NVP-HSP990 and hyperthermia before irradiation with 4 Gy caused a delayed acumulation of cells in the G2/M phase. The strongest effect was detected 24 hours after irradiation. Three to five days after the irradiation teatment, we observed a strong increase of the number of cells with morphological signs of mitotic catastrophe (micro- and multinucleated cells) [34,35]. The number of apoptotic cells also increased. As large numbers of necrotic cells (taking up PI) were not found, this suggests that mitotic catastrophe constitutes a prelude to apoptotic cell death. Similar findings were reported for a novel small molecule inhibitor that lowers the threshold for Hsf1 (Heat shock factor protein 1) activation [36]. The inhibition enhanced thermal sensitivity and significant thermal radiosensitization followed by loss of mitochondrial potential and mitotic catastrophe in HT29 colon carcinoma cells.

## Conclusion

Taken together, our study shows that NVP-HSP990, a fully synthetic, orally available Hsp90 inhibitor exibits strong anti-tumor effects on U251 human glioblastoma and MIA PaCa-2 pancreatic carcinoma cells through an increase of sensitivity towards heat and ionising irradiation. Further preclinical studies are warranted to clarify the complex mechanisms of its action and to explore the therapeutic potential of this approach *in vivo*.

## Competing interests

Authors certify that there is no actual or potential conflict of interest in relation to this article.
